# Psychometric study of the Awareness of Age-Related Change (AARC) Short Scale translated to Portuguese, applied to Brazilian older adults

**DOI:** 10.1590/1980-57642021dn15-020011

**Published:** 2021

**Authors:** Anita Liberalesso Neri, Hans-Werner Wahl, Roman Kaspar, Manfred Diehl, Samila Sathler Tavares Batistoni, Meire Cachioni, Mônica Sanches Yassuda

**Affiliations:** 1 Graduate Program of Gerontology, School of Medical Sciences, Universidade Estadual de Campinas - Campinas, SP, Brazil.; 2 Institute of Psychology, Heidelberg University - Heidelberg, Germany.; 3 Cologne Center for Ethics, Rights, Economics and Social Sciences of Health, University of Cologne - Cologne, Germany.; 4 Department of Human Development and Family Studies, Colorado State University - Fort Collins, USA.; 5 School of Arts, Sciences and Humanities, Universidade de São Paulo - São Paulo, SP, Brazil.

**Keywords:** reliability and validity, self-perception, attitude, aged, reprodutibilidade dos testes, autopercepção, atitude, idoso

## Abstract

The concept Awareness of Age-Related Change (AARC) is defined as a person’s awareness that their behavior, level of physical, cognitive and social performance, and ways of experiencing life have changed as a consequence of having grown older, and not because of disease.

**Objective:**

A psychometric study investigating evidence of construct validity and internal consistency of the Portuguese version of the AARC Short Scale was carried out.

**Method:**

A convenience sample of 387 individuals aged≥60 years with no deficit suggestive of dementia were recruited at venues frequented by older persons and at households. Participants answered the Portuguese version of the scale, along with questionnaires collecting sociodemographic and frailty variables and self-rated health based on personal criteria and relative to peers.

**Results:**

Exploratory and confirmatory factorial analyses derived a structure with two orthogonal factors representing the latent variables gains and losses, invariant for age group, thus replicating the original scale. The factors explained a large proportion of item variability (58.6 to 51.8%) and exhibited high loadings (0.886 to 0.432) and good communality [0.787 for item 4 (*better sense of what is important*) and 0.369 for item 6 (*less energy*)]. The hypotheses of covariance between the new instrument and the parallel measures of frailty and self-rated health were confirmed. The levels of internal consistency were high (α>0.700).

**Conclusion:**

Evidence confirmed the factor and convergent (construct) validity and internal consistency of the new scale in Portuguese.

## RESUMO.

O reconhecimento de ganhos e perdas associados ao envelhecimento desempenha papel regulador em relação ao *self* e às atitudes sociais quanto à velhice.

**Objetivo:**

Investigar indicadores de validade de construto, consistência interna e validade convergente da *Awareness of Age-Related Change Short Scale* (AARC) traduzida para o português.

**Método:**

Um total de 387 indivíduos de 60 anos ou mais compuseram amostra de conveniência que respondeu à versão do estudo *AARC Short Scale* em português, e a medidas de fragilidade, autoavaliação de saúde e caracterização sociodemográfica. As explorações de validade de construto foram mediadas por análises fatoriais exploratórias (com rotação Varimax e oblíqua/Promax), e confirmatórias (método de equações estruturais para variáveis latentes); as de consistência interna, pelo teste de Cronbach e as de validade convergente, pelas correlações entre os itens da escala, fragilidade e saúde autorreferida.

**Resultados:**

A estrutura de dois fatores, ganhos e perdas, as cargas e a comunalidade dos itens da escala em português foram altas e similares nas análises fatoriais exploratórias e confirmatórias, e similares à versão original. Foi rejeitada a hipótese de correlação entre os fatores e confirmada a de invariância do construto às idades; consistência interna da escala e dos fatores indicada por índices α>0,700; fragilidade e saúde autorreferida correlacionaram-se negativamente com a pontuação na escala e em ganhos, e positivamente em perdas.

**Conclusões:**

Foram observadas evidências de validade de construto, consistência interna e validade convergente da versão do *AARC Short Scale* em português, para aplicação em amostras com características similares às desse estudo.

## INTRODUCTION

The concept Awareness of Age-Related Change (AARC) was introduced in the lifespan psychology and aging literature by Diehl and Wahl,^1^ who defined it as a person’s awareness that his/her behavior, level of physical, cognitive and social performance, and ways of experiencing life have changed as a consequence of having grown older,^1^ ^,^ ^2^ and not because of disease. The construct aims to enrich the overarching area of subjective aging which also includes concepts such as self-perception of aging,^3^ age identity,^4^ attitudes toward aging^5^ and aging stereotype.^6^ Many of these concepts operate mainly at a pre-conscious level, whereas AARC is seen mainly as a conscious reflection upon one’s aging.^1^ ^,^ ^2^ ^,^ ^3^ ^,^ ^7^

In congruence with its ties to the lifespan paradigm in psychology,^8^ the AARC concept goes beyond the common positive-negative polarity.^1^ ^,^ ^2^ Gains and losses can be experienced concomitantly, within the same or different behavioral domains, such as health and physical functioning, cognitive functions, interpersonal relationships, social cognitive functioning, socioemotional functioning, and lifestyle and social engagement.^8^ Evidence supporting that these behavioral domains are the source for most of adults’ subjective aging experiences was obtained in several focus groups^9^ and in a daily diary study with community-residing older adults in Germany.^10^

The notion of gains and losses occurring over chronological time organizes and guides the construction of information on the self, such as the subjective experience of aging.^11^ ^,^ ^12^ In the collective sphere, the notion of gains and losses plays a central role, shaping normative age expectations^13^ and attitudes toward aging,^14^ reinforcing stereotypes of aging,^15^ and mediating the effects of age stereotypes on physical and mental health.^16^

A major line of existing research on awareness of age-related change has addressed the link between AARC and several physical and mental health.^17^ A number of studies support consistently that particularly heightened AARC-Loss is linked over time to lowered functional health, lowered psychological well-being, and heightened depressive symptoms. AARC-Gains have been found to predict positive developmental outcomes such as increased well-being over time, but effect sizes overall tended to be lower compared to AARC-Losses.^18^ ^,^ ^19^ ^,^ ^20^

AARC measurements started by generating a large item pool related to subjective aging experiences leading to an instrument with 189 items with good psychometric properties, in samples of 40 years and older, but needing a rather long assessment time. Therefore, the psychometric properties of a reduced 50-item version was successfully examined in a sample of German and North American participants 40 years and older. Making use of exploratory and confirmatory factor analyses, it was confirmed that the hypothesized two latent factor solution in terms of AARC-Gains and AARC-Losses in all five domains showed sufficient reliability.^21^ A third step created an instrument based on a 10-item short form. The rationale behind this was that beyond focusing the AARC domains, the instrument would be helpful for use in large-scale surveys as well as the integration of the instrument in existing studies worldwide, thus also allowing to serve inter-cultural comparative purposes in the longer run. Item selection was based on Item Response Theory procedures and the 2-factor solution was again clearly confirmed. In addition, measurement invariance across age groups (40-69, 70-79 and 80+ years) was largely supported as well as concurrent and discriminant validity by testing inter-relations with developmental outcomes such as subjective aging, well-being, and health measures.^22^

Given the interest to learn about the awareness of age-related change in different cultures and countries, an important gap is the dearth of theoretical approaches and research instruments in the international, and particularly in the Portuguese-speaking research community that investigates aging. Thus, evidence-based social and old age-related policy aimed to improve health, well-being, images and experiences of aging are to a large extent limited. Older adults in Portuguese-speaking countries, such as those in Brazil, Portugal, and former Portuguese colonies in Africa and Asia, would benefit greatly from new knowledge and attitudes in relation to aging derived from the use of internationally established subjective aging measurement devices. The short AARC is being used in large survey studies such as the North-Rhine Westphalia 80+, in Germany,^23^ and the PROTECT study in the UK with individuals aged 50+.^24^ Hence, a Portuguese language-based short AARC instrument also has potential for profound comparative approaches internationally.

As a result of such reasoning, a psychometric study based on data from a sample of Brazilian older adults was conducted, which was aimed at investigating indicators of construct validity (dimensionality in particular), internal consistency and convergent validity of the AARC (Awareness of Age-Related Change) Short Scale translated from English and semantically adapted to Portuguese.

## METHODS

### Participants

The study drew on a database of a convenience sample of 387 older adults who took part in a survey collecting opinions and expectations of older adults on the physical and social environment of the city in which they lived. Recruitment was performed by professionals at locations frequented by older adults and at participants´ homes; through friends and acquaintances using the snowball techniques; and via newspapers, leaflets, talks, TV and social networks. The following inclusion criteria were applied: age≥60 years; score>cut-off points in the Mini-Mental State Examination, a cognitive screening test administered at the start of the interview^25^ ^,^ ^26^ (17 for those who never attended school; 22 for those with 1 to 4 years and 24 for those with 5 to 8 years of schooling, and 26 for those who had attended school for 9 or more years),^27^ and fully answered scales applied for the study.

### Bioethical approach

All participants signed a free and informed consent form outlining the conditions of their participation and the ethical principles observed by the research team. The study project was approved by the Research Ethics Committee of the Universidade Estadual de Campinas (registration No. 1.902.178 and Certificate for Ethical Appreciation No. 62580416.7.0000.5404).

### Measures

The 10-item AARC short scale^22^ was translated into Portuguese according to the guidelines of Beaton et al.^28^ for the process of translation and cross-cultural adaptation of self-report measures. The team comprised the following members: two Brazilian psychologists who were native Portuguese speakers and fluent in English (T1 and T2), one blinded and the other not blinded to the content and structure of the instrument; a Brazilian psychologist who was a native Portuguese speaker, fluent in English and informed about the scale (T3); and two professional blinded translators, one who was a native Portuguese speaker and fluent in English and the other a native English speaker and fluent in Portuguese (T4 and T5). These professionals, first working independently, then in pairs and lastly as a panel, produced translations, back translations and a cultural-semantic adaptation of the instrument. T3 acted as the mediator in the comparisons and syntheses of the translations and back translations, as well as in the cultural adaptation process. Upon conclusion of this process, the experts suggested designating the instrument in Portuguese as the *Escala de Consciência das Mudanças Associadas ao Envelhecimento -* CMAE-10.

To facilitate the answering of the scale by low-educated individuals, the items expressing gains were placed first, in the order 2, 4, 1, 5 and 9, renumbered 1 to 5; these were followed by items associated with losses, in the order 7, 3, 8, 10 and 6, renumbered 6 to 10. In the original version, the 10 items are scored on a Likert-type scale, from 1 (not at all) to 5 points (very much). For the Brazilian version, a 4-point response format was elected (0=not at all, 1=a little, 2=much and 3=very much). An instruction on the logic of the responses was devised, comparing the meaning of extreme versus intermediate points. Furthermore, a statement was provided emphasizing there are no right or wrong answers and that the aim was merely to gather participant experiences. For each item, respondents were asked to consider the following stem: “With my increasing age, I realize that…”.

**Frailty according to self-report.** A questionnaire consisting of four dichotomous items (unintentional weight loss, weakness, slow walking speed and low physical activity in the last year) and two scaled items (exhaustion always or almost always in the last week),^29^ validated using the phenotype model of frailty devised by Fried et al.,^30^ were applied.

**Self-reported health.** On the basis of a scaled item with five intensities (1=very poor; 2=poor, 3=moderate, 4=good and 5=very good), participants were asked to rate their own health according to non-specified criteria and relative to the health of peers.^31^ ^,^ ^32^ ^,^ ^33^

**Sociodemographics. Age** was determined on the basis of date of birth; **sex** options were male or female; number of **years of education** was determined by the question: “up to what grade did you go to school?” Five questions were addressed to obtain additional data on the characteristics of the sample, but they were not part of the data analyses: race/skin color, living arrangement, employment, retirement and home ownership status.

### Statistical methods

Responses on the CMAE-10 scale were used as a basis for two types of psychometric analyses: construct or internal validity, which included the subcomponents factor validity and convergent validity, and internal consistency or reliability of non-validity. Construct validity was tested by performing exploratory factor analysis (EFA) using Varimax rotation, which assumes independence between factors, and with oblique Promax rotation, a counter test that assumes correlation between the items of the factors. Confirmatory factor analyses (CFA) were performed to assess the factor structures resulting from EFA, based on the structural equations model for latent variables.^34^ Due to the new study context provided by the Portuguese translation and the region of Brazil, we regarded both EFA and CFA as important.

The sample was divided twice to carry out the factor analyses. First, randomly, and second by age criteria, to test the hypothesis of invariance of the composition of the factors for age group. The first division yielded two randomized subsamples containing 194 and 193 participants, respectively. The data were analyzed using EFA for the first sample and CFA for the second. The age subgroups were formed by stratifying the sample into 4 groups: 2 groups for individuals aged 60-69 years, containing 118 and 117 participants, respectively, and 2 for those aged 70-80+, each containing 76 participants. The data for the first subgroup aged 60-69 years and the first subgroup aged 70-80+ were both submitted to EFA, while the two parallel subsamples were submitted to CFA.

Prior to performing EFA, degree of adequacy of the subsample for factor analysis was tested using the Kaiser-Meyer-Olkin (KMO) Measure of Sampling Adequacy (MSA). The number of factors to be extracted, with eigenvalues >1, was decided on the basis of the Scree Test. The CFA models comprised fixed parameters (factor loadings=zero) and free parameters to be estimated (factor loadings≠zero). The following measures of adequacy of fit were adopted: chi-square test for the matrices of covariance of the estimated sample and real sample; goodness of fit index (GFI); adjusted GFI for degrees of freedom (AGFI); standardized root mean square residual (SRMR); root mean square error of approximation (RMSEA); Bentler’s comparative fit index (CFI), and Bentler & Bonett’s non-normed fit index (NNFI). Wald’s test was used to propose item exclusion and Lagrange multipliers (p<0.05) was used for item reallocation.

The scale and its factors were tested for internal consistency using Cronbach’s alpha: coefficient α>0.70 indicated high internal consistency, α=0.30-0.69 moderate, and α<30 low internal consistency.

The investigation of indicators of convergent validity was based on Spearman’s correlation test between values of the items from the CMAE-10 scale and values of frailty and self-rated health measures, which are theoretically associated with the gains and losses construct. The following hypotheses were tested: (a) the lower the number of frailty criteria, the higher the score on the CMAE-10 scale and the greater the gains factor score; (b) the higher the number of frailty criteria, the lower the self-rated health score; (c) the greater the number of frailty criteria, the higher the losses score; and (d) the higher the number of frailty criteria, the lower the gains score.

## RESULTS

The sample that produced the data for the psychometric studies comprised individuals who were mostly women, aged 60‒70 years, and had education ≤4 years; these persons were white, married, homeowners, retired and not engaged in paid employment. Interviews were conducted in public parks (37.3%), homes (22.8%), primary care clinics (14.2%), day-care centers (10.6%), voluntary organizations (7.8), public streets (5.7%) and the workplace (1.6%). Most respondents scored higher for gains than losses, high for frailty, and very high for self-rated health (Table 1).

**Table 1.** Sample characteristics. CMAE-10, Psychometric Study. Brazil, 2020.

	Total	n (%)	Me (SD)	Min	Md	Max
Age	387		67.9 (5.6)	60.0	67.0	63.0
60-70 years 71-80+ years		235 (60.7) 152 (39.2)				
Sex	387					
Male Female		156 (40.3) 231 (59.7)				
Education	386		5.4 (4.1)	0.0	4.0	15.0
>5 years 5-8 years 9-11 years ≥12 years		254 (65.8) 70 (18.2) 33 (85.5) 29 (7.5)				
Marital status	384					
Married Widow(er) Single Divorced		242 (63.0) 80 (20.9) 23 (6.0) 39 (10.1)				
Living arrangements	381					
Living alone Living with partner Other arrangement		54 (14.2) 241 (63.2) 86 (22.6)				
Homeowner	384					
Yes No		304 (79.2) 80 (20.8)				
Working	377					
Yes No		73 (19.3) 304 (80.7)				
Retired	374					
Yes No		259 (69.5) 114 (30.5)				
Frailty criteria	386		2.6 (1.5)	0.0	2.0	5.0
0 1 2 3 4 5		70 (18.1) 67 (17.3) 86 (22.3) 78 (20.2) 64 (16.6) 21 (5.5)				
Self-rated health	385		3.8 (0.8)	1.0	2.0	5.0
Very poor Poor Fair Good Very good		2 (0.5) 11 (2.8) 121 (31.5) 184 (47.8) 67 (17.4)				
Self-rated health relative to peers	379		3.7 (0.8)	1.0	4.0	5.0
Worse Same Better		26 (6.8) 112 (29.6) 164 (43.3)				
Much better		77 (20.3)				
CMAE-10 Total CMAE-10. Gains CMAE-10. Losses	387		20.5 (4.2) 11.3 (2.8) 5.8 (3.4)	0.0 0.0 0.0	20.0 11.0 5.0	30.0 30.0 15.0

SD: standard deviation.

The process of translation and semantic-cultural adaptation of the AARC Short Scale for Portuguese yielded the translation given in Chart 1, together with the original items and description of their content.

**Chart 1.** Content and equivalence of the AARC Short Scale and *Escala CMAE-10*, and positions measures of the items in a Brazilian sample with aged persons, 2020.

Domains of functioning	Nature of change	AARC Short Scale - original version	***Escala CMAE-10 - Portuguese* version**	n	Me (SD)	Min.	Md	Max.
PHF	Gain	1. I pay more attention to my health.	1. *Presto mais atenção à minha saúde.*	387	2.2 (0.7)	0. 0	2.0	3.0
COG	Gain	2. I have more experience and knowledge to evaluate things and people.	2. *Tenho mais experiência e conhecimento para avaliar coisas e pessoas.*	387	2.2 (0.8)	0.0	2.0	3.0
IRS	Gain	3. I appreciate relationships and people much more.	3. *Valorizo muito mais as relações sociais e as pessoas.*	387	2.4 (0.6)	0.0	2.0	3.0
SEF/SCF	Gain	4. I have a better sense of what is important to me.	4. *Tenho mais noção do que é importante para mim.*	387	2.4 (0.6)	0.0	2.0	3.0
LSI	Gain	5. I have more freedom to live my days the way I want.	5*.Tenho mais liberdade para viver meus dias do jeito que quero.*	387	2.2 (0.8)	0.0	2.0	3.0
PHF	Loss	6. I have less energy.	6. *Tenho menos energia.*	387	1.5 (0.8)	0.0	2.0	3.0
COG	Loss	7. My mental capacity is declining.	7. *Minha capacidade mental está declinando.*	387	1.2 (0.9)	0.0	1.0	3.0
IRS	Loss	8. I feel more dependent on the help of others.	8. *Sinto-me dependente da ajuda dos outros.*	387	0.8 (1.0)	0.0	0.0	3.0
SEC/SCF	Loss	9. I find it harder to motivate myself.	9. *Sinto que está mais difícil me motivar.*	387	1.1 (1.0)	0.0	1.0	3.0
LSI	Loss	10. I have to limit my activities.	10. *Tenho que limitar minhas atividades.*	387	1.1 (0.9)	0.0	1.0	3.0

Me = Mean; SD = Standard Deviation; Min = Minimum; Md = Median; Max = Maximum. DP. PHF: physical health and functioning; COG: cognitive functioning; IRS: interpersonal relationships; SEF/SCF: socioemotional functioning/socio-cognitive functioning; LSI: life-style and involvement.

The first half of the randomly selected sample had a MAS/KMO=0.78, and thus proved suitable for application of the EFA. On the basis of the selection criteria of factors with an eigenvalue >1 and of the Scree Test, 2 factors explaining 55.4% of the variance of the items were selected. Varimax rotation was applied to the data. Factor 1 (Gains) encompassed items 1, 4, 2, 3, and 5, with factor loadings of 0.82 to 0.65. Factor 2 (Losses) consisted of items 10, 9, 8, 7 and 6 and had factor loadings of 0.81 to 0.48. Item 4 had the greatest communality (67.4%) and item 6 (35.5%) the lowest. The data were rotated for a second time using the oblique strategy. The hypothesis of the existence of correlation was rejected, where r=0.110. The data from the 2nd randomized subsample were analyzed using CFA. Two sequences of item reallocation were performed, resulting in a return to the first factorial solution, which confirmed the data from the EFA of the 1st randomized subsample (Table 2).

**Table 2.** Results of the first factorial exploratory and confirmatory analyses (FEA and FCA) over the data derived from the sample of aged Brazilian persons who had answered to the CMAE-10, 2020.

Factors	Domains	Items	Statements	1ª FEA. 1^st^ sub sample: aged 60 to 69*		1^st^ FCA. 1st sub sample: aged 70 to 80+**	
				Loads	Communality	Loads	t-values
	SCF	4	I have a better sense of what is important to me.	0.820	0.674	0.823	12.62***
	PHF	1	I pay more attention to my health.	0.751	0.565	0.607	8.54***
1. Gains	COG	2	I have more experience and knowledge to evaluate things and people.	0.745	0.558	0.730	10.78***
	IRS	3	I appreciate relationships and people much more.	0.717	0.528	0.664	9.55***
	LSI	5	I have more freedom to live my days the way I want.	0.652	0.427	0.601	8.45***
	LSI	10	I have to limit my activities.	0.812	0.659	0.652	8.63***
	SCF	9	I find it harder to motivate myself.	0.791	0.627	0.767	10.22***
2. Losses	IRS	8	I feel more dependent on the help of others.	0.780	0.609	0.475	6.07***
	COG	7	My mental capacity is declining.	0.723	0.535	0.617	8.13***
	PHF	6	I have less energy.	0.478	0.454	0.340	4.22***

*Parameters to FEA: Kaiser-Mayer-Olkin MSA=0,773; Varimax rotation; eigenvalues >1; total variability of data: 58.6%.

**Measures of goodness of fit indexes to the FCA: chi-square=27.21; chi-square DF=26; chi-score ratio=1.05; p-value=0.3986. Goodness of Fit Index (GFI)=0.9483; GFI Adjusted for Degrees of Freedom (AGFI)=0.9104; Bentler's Comparative Fit Index (CFI)=0.9952; Bentler & Bonett's (1980) (NNFI)=0.9933; Standardized Root Mean Square Residual (SRMR)=0.0588; RMSEA Estimate=0.0200/CI90% 0.0372‒0.0900.

***significant t-values to the standardized load of the items >1.96.

PHF: physical health and functioning; COG: cognitive functioning; IRS: interpersonal relationships; SEF/SCF: socioemotional functioning/sociocognitive functioning; LSI: life style and involvement.

From the analyses of the internal consistency of the CMAE-10 scale resulted: in the 1st AFE, α=0.777 for Factor 1 (Gains) and α=0.777 for Factor 2 (Losses), for Varimax or Oblique rotation; in the 2nd AFE, α=0.849 in Factor 1 and 0.767 in Factor 2, for any type of rotation; in the 1st AFC: α=0.817 for Gains and α=0.702 for Losses (with Varimax rotation), and α=0.790 (for Gains) and α=0.695 (for Losses), for Oblique rotation (Tables 2 and 3).

For both EFA and CFA, the results for the 60-69 years subsample were similar to those of the randomized subsamples. The EFA of the data from the 1st 70-80 years subsample showed that factor 1 retained the losses and excluded item 6, while factor 2 included the gains. This factorial solution was not maintained on the CFA applied to the data from the 2nd 70-80+ years subsample and item 6 was reallocated to factor 2. The hypothesis of a correlation between the factors was rejected (r=0.058) and the hypothesis of invariance of the construct for the age groups was confirmed (Table 3).

**Table 3.** Results from the second exploratory and confirmatory analyses (FEA and FCA) over the data derived from the sample of aged Brazilian persons who had answered to the CMAE-10, 2020.

Factors	Domains	Items	Statements	2^nd^ FEA. 1^st^ sub sample: aged 60 to 69*		2^nd^ FCA. 1^st^ sub sample: aged 70 to 80+**	
				Loads	Communality	Loads	t-values
	SCF	4	I have a better sense of what is important to me.	0.886	0.787	0.817	7.87***
	LSI	3	I appreciate relationships and people much more.	0.865	0.489	0.646	5.32***
1. Gains	PHF	1	I pay more attention to my health.	0.844	0.713	0.669	6.06***
	COG	2	I have more experience and knowledge to evaluate things and people.	0.764	0.585	0.734	7.87***
	IRS	5	I have more freedom to live my days the way I want.	0.745	0.557	0.603	5.79***
	LSI	10	I have to limit my activities.	0.828	0.686	0.862	8.23***
2. Losses	SEF	9	I find it harder to motivate myself.	0.785	0.619	0.742	6.82***
	IRS	8	I feel more dependent on the help of others.	0.756	0.584	0.478	4.04***
	COG	7	My mental capacity is declining.	0.670	0.470	0.742	4.99***
	PHF	6	I have less energy.	0.524	0.369	0.862	4.65***

*Parameters of FEA: Kaiser-Mayer-Olkin MSA=0.773; Varimax rotation; eigenvalues >1; total variability of data: 78.7%.

**Measures of goodness of fit index to CFA: chi-square=46.25; chi-square DF=34; p-value=0.0784; chi-score ratio=1.36; Goodness of Fit Index (GFI)=0.9049; GFI Adjusted for Degrees of Freedom (AGFI)=0.8462; Bentler's Comparative Fit Index (CFI)=0.9461; Bentler & Bonett's (1980) (NNFI)=0.9286; Standardized Root Mean Square Residual (SRMR)=0.0683; RMSEA Estimate=0.093/CI90% 0.0000‒0.1158.

***t-values significant of the standartized items loads >1.96.

PHF: physical health and functioning; COG: cognitive functioning; IRS: interpersonal relationships; SEF/SCF: socioemotional functioning/sociocognitive functioning; LSI: life style and involvement.

For the total sample and age subsamples, negative correlations between frailty score and CMAE-10 scale scores and in gains were found. The inverse pattern was identified for losses: the higher the frailty score, the higher the losses score. The higher and more positive self-rated health, the higher the score was on the scale and in gains; the more negative the result, the higher the losses score was. This pattern repeated for self-rated health compared with peers of the same age, with systematically lower correlations, except for losses among individuals aged 70 years and over (Table 4).

**Table 4.** Correlations between the sample scores on the CMAE-10 Scale, frailty, self-rated health, and self-rated health in comparison with others of the same age, 2020.

		Frailty criteria	Self-rated health	Self-rated health in comparison with others of the same age
Total sample				
CMAE-10 Total	r p n	**-0.352** <0.0001 386	**0.323** <0.0001 385	**0.316** <0.0001 379
Factor 1. Gains	r p n	**-0.113** 0.0256 386	**0.221** <0.0001 385	**0.198** <0.0001
Factor 2. Losses	r p n	**0.353** <0.0001 386	**-0.258** <0.0001 385	**-0.254** <0.0001 379
60 to 69 years				
CMAE-10 Total	r p n	**-0.346** <0.0001 234	**0.343** 0.0001 233	**0.292** <0.0001 231
Factor 1. Gains	r p n	-0.094 0.150 234	**0.228** 0.004 233	**0.146** 0.0261 231
Factor 2. Losses	r p n	**0.401** <0.0001 234	**-0.286** <0.0001 233	**-0.262** <0.0001 231
70 to 80 years and above				
CMAE-10 Total	r p n	**-0.328** <0.0001 152	**0.291** 0.0003 152	**0.352** <0.0001 148
Factor 1. Gains	r p n	-0.148 0.067 152	**0.121** 0.0007 152	**0.283** 0.0005 148
Factor 2. Losses	r p n	**0.259** <0.0001 152	**-0.210** <0.0009 152	**-0.225** 0.0059 148

## DISCUSSION

We investigated the evidence of the construct validity of the AARC Short Scale in Portuguese applied to a sample of Brazilian older adults aged 60 years and older. Strong support for construct validity was found. The items of the factors exhibited high loadings and good communality, proved independent from one another and representative of the conceptually predicted latent constructs AARC-Gains and AARC-Losses, which also proved invariant for age group (factor validity). Hence, the factor structure of the scale in Portuguese proved similar to that of the original scale.^22^ The expectation of covariance between the new instrument and measures of frailty and self-reported health according to previous work conducted in middle- development countries^32^ ^,^ ^33^ ^,^ ^34^ ^,^ ^35^ ^,^ ^36^ ^,^ ^37^ ^,^ ^38^ was also confirmed. The levels of internal consistency were high, further supporting the evidence of construct or internal validity.

The participants scored higher for gains than for losses, an expected result given that most respondents were aged 60-69 years, active and scored high or very high on self-rated health based on personal and social comparison criteria. However, the 42.3% rate of frailty was surprisingly high, a level typically seen in middle-development country samples of old-old, institutionalized, ill, functionally dependent, ailing or inactive elderly scoring on three or more frailty criteria.^35^ ^,^ ^36^ ^,^ ^37^ ^,^ ^38^ However, participants’ responses may have been more influenced by the research setting (could it seem like a good opportunity to complain about health problems?), for concerns about own aging,^3^ ^,^ ^38^ doubts regarding age identification,^38^ ^,^ ^39^ ^,^ ^40^ aging stereotypes^14^ ^,^ ^16^ and uncertainty regarding the age bracket defining older persons (or age categorization),^39^ than by actual loss of energy and independence. In accordance with the literature,^16^ ^,^ ^17^ ^,^ ^18^ ^,^ ^19^ ^,^ ^20^ we hypothesized that losses scores would be positively correlated with negative ratings of own health, as well as gains positively correlating with positive scores, data that were confirmed by the study results.

The main limitations of the present study were that a convenience sample was used and that confirmatory factor analyses were not cross-validated.^34^ Another limitation was the fact that the invariance of the factors for age was suggested by comparing data between adjacent age groups and not by longitudinal data. In addition, the variables used for the convergent validity study did not belong to the same conceptual universe of the AARC. However, the self-report measure of frailty was validated against two objective criteria (loss of strength and slowed gait) and three self-report criteria of conditions that respondents were able to observe objectively in themselves (weight loss, intense exhaustion and low level of physical activity).^29^ ^,^ ^30^ There is a large body of literature supporting the validity of self-rated health for objective variables.^32^ ^,^ ^33^

The geographic and socioeconomic heterogeneity of the sample, together with the detailed description of its characteristics, further contribute to adaptation of the scale for use in other samples. Although not probabilistic, the sample was fairly representative of the city populations aged 60 and over, living in small to medium-sized cities, which are the majority of Brazilian cities.

This study makes a useful innovative contribution to the field. As indicated by the psychometric evidence of internal or construct validity (dimensionality in particular), age invariance and internal consistency, the Brazilian version of the AARC Short Scale seems to be similar to the original in English. The Brazilian version should now be assessed to determine whether cultural adaptation is needed for use in other Portuguese-speaking countries.

Given its content and ease of application, the scale has clinical, educational and social utility, where attitudinal changes self-directed or directed toward others regarding aging are required. In social, organizational and public policy settings, data produced by the scale can help sensitize individuals and institutions to better cater to the unmet needs of the oldest population and make concerted efforts to counter ageism, a phenomenon that has a major negative impact on low-income, low-educated individuals who age in societies with poor economic organization and inherent inequality.
